# Development and Validation of a Simple-to-Use Nomogram for Predicting the Upgrade of Atypical Ductal Hyperplasia on Core Needle Biopsy in Ultrasound-Detected Breast Lesions

**DOI:** 10.3389/fonc.2020.609841

**Published:** 2021-03-31

**Authors:** Yun-Xia Huang, Ya-Ling Chen, Shi-Ping Li, Ju-Ping Shen, Ke Zuo, Shi-Chong Zhou, Cai Chang

**Affiliations:** ^1^ Department of Ultrasonography, Fudan University Shanghai Cancer Center, Shanghai, China; ^2^ Department of Oncology, Shanghai Medical College, Fudan University, Shanghai, China; ^3^ Department of Breast Surgery, Fudan University Shanghai Cancer Center, Shanghai, China; ^4^ Department of Pathology, Fudan University Shanghai Cancer Center, Shanghai, China

**Keywords:** atypical ductal hyperplasia, breast, ultrasound, upgrade, prediction

## Abstract

**Background:**

The rate of carcinoma upgrade for atypical ductal hyperplasia (ADH) diagnosed on core needle biopsy (CNB) is variable on open excision. The purpose of the present study was to develop and validate a simple-to-use nomogram for predicting the upgrade of ADH diagnosed with ultrasound (US)-guided core needle biopsy in patients with US-detected breast lesions.

**Methods:**

Two retrospective sets, the training set (*n* = 401) and the validation set (*n* = 186), from Fudan University Shanghai Cancer Center between January 2014 and December 2019 were retrospectively analyzed. Clinicopathological and US features were selected using univariate and multivariable logistic regression, and the significant features were incorporated to build a nomogram model. Model discrimination and calibration were assessed in the training set and validation set.

**Results:**

Of the 587 ADH biopsies, 67.7% (training set: 267/401, 66.6%; validation set: 128/186, 68.8%) were upgraded to cancers. In the multivariable analysis, the risk factors were age [odds ratio (OR) 2.739, 95% confidence interval (CI): 1.525–5.672], mass palpation (OR 3.008, 95% CI: 1.624–5.672), calcifications on US (OR 4.752, 95% CI: 2.569–9.276), ADH extent (OR 3.150, 95% CI: 1.951–5.155), and suspected malignancy (OR 4.162, CI: 2.289–7.980). The model showed good discrimination, with an area under curve (AUC) of 0.783 (95% CI: 0.736–0.831), and good calibration (*p* = 0.543). The application of the nomogram in the validation set still had good discrimination (AUC = 0.753, 95% CI: 0.666–0.841) and calibration (*p* = 0.565). Instead of surgical excision of all ADHs, if those categorized with the model to be at low risk for upgrade were surveillanced and the remainder were excised, then 63.7% (37/58) of surgeries of benign lesions could have been avoided and 78.1% (100/128) malignant lesions could be treated in time.

**Conclusions:**

This study developed a simple-to-use nomogram by incorporating clinicopathological and US features with the overarching goal of predicting the probability of upgrade in women with ADH. The nomogram could be expected to decrease unnecessary surgery by nearly two-third and to identify most of the malignant lesions, helping guide clinical decision making with regard to surveillance *versus* surgical excision of ADH lesions.

## Introduction

Approximately 20% of the suspicious breast lesions diagnosed by core needle biopsy (CNB) demonstrate atypical ductal hyperplasia (ADH) (1). Morphologically, ADH is similar to low-grade ductal carcinoma *in situ* (DCIS) and their differentiation is based only on the lesion size whereby a lesion that restricted to ducts and ductules and ≤2 mm in maximum diameter is classified as ADH and a larger lesion is classified as low-grade DCIS. Therefore, potential for underestimation of malignant lesion may exist in ADH lesions obtained with CNB. For this reason, the current standard of care is to excise ADH found at percutaneous biopsy to exclude co-existing cancer even if the lesions seems to be completely excised by vacuum-assisted biopsy (2). The underestimated rates have been reported to range from 0 to 84%, although most studies found upgrade rates of approximately 25% (2, 3). Therefore, surveillance instead of surgical excision might be appropriate in patients with low risk of upgrade (4–6). This emphasizes the need to find approaches to identify those women who are more likely to have a cancer, which may contribute to the early detection of breast cancer. Thereby, low-risk patients will be advised to safely forgo surgical treatment (7).

Previous studies have attempted to identify features which are correlated with the likelihood of ADH upgrade, including age, lesion size, type of biopsy gauge, number of cores, and extent of ADH (5, 6, 8). However, none of these features can reliably stratify patients into different treatment subgroups (8–10). The diversity or even conflict in risk factors might be due to the study design because the investigated potential risk factors varied widely and most of the studies were single institution studies with a limited number of cases. Some studies have suggested that combining imaging features with biopsy and clinical characteristics maybe necessary for improving the preoperative prediction of final pathological diagnosis for ADH (6, 8, 11–14). The ultrasound (US) features such as margin, size of the lesions, and echogenicity features, are vital for the detection and discrimination of benign and malignant lesions (15), and also help in prediction of axillary lymph node metastasis in breast cancer (16). US has been widely used in imaging-guided breast CNB, especially in developing countries, with the advantage of real-time visualization, less expensive, not using ionizing radiation, and not limited to breast density (15, 17). However, only a limited number of studies have investigated the use of US features for predicting the upgrade of ADH (18). Therefore, the main aim of this study was to ascertain clinicopathological and US features with the goal of developing and validating a nomogram that will be able to predict the risk of pathologic upgrade in patients with ADH diagnosed with CNB.

## Methods

### Patient Selection

This retrospective study was approved by the review board of Fudan University Shanghai Cancer Center. The training set for this study comprised of 401 patients who were diagnosed with ADH by using US-guided core biopsy and underwent surgical resection at our institution from January 2014 to December 2018. A validation set of 186 consecutive patients was then established from January to December 2019 using the same criteria. All patients underwent percutaneous US-guided CNB using the 14-gauge automated gun method (Gallini, Mantova, Italy or Bard Technologies, Covington, USA), and approximately five cores were taken from each lesion by the surgeon.

### Inclusion and Exclusion Criteria

**Figure 1:**
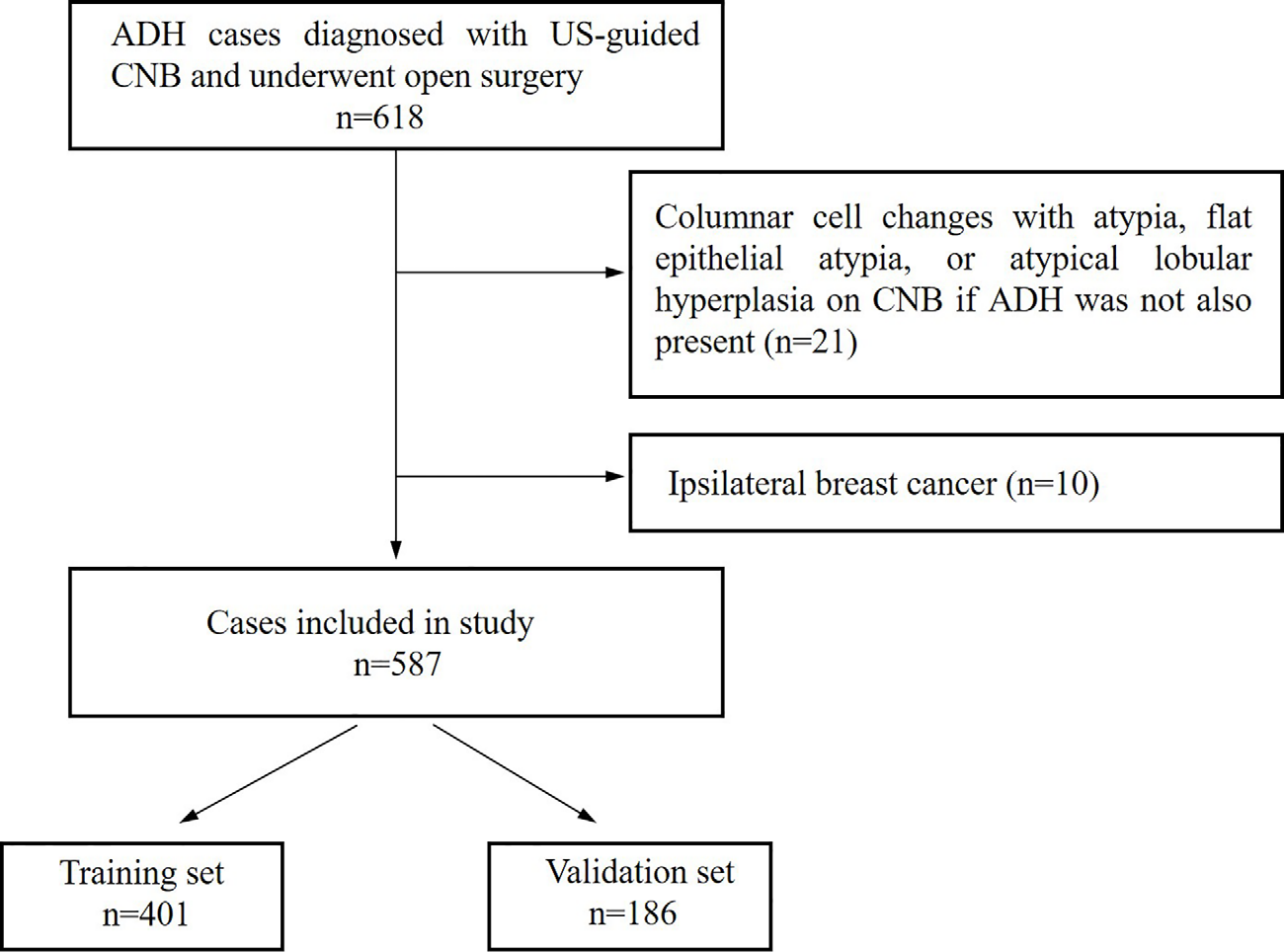
Flow chart of cases selection from atypical ductal hyperplasia (ADH) cases diagnosed between January 2014 and December 2019.

This study included patients who were diagnosed with ADH using US-guided CNB, without DCIS or invasive carcinomas in the same biopsy, and no lymph node metastases found preoperatively. We excluded lesions diagnosed as columnar cell changes with atypia, flat epithelial atypia, or atypical lobular hyperplasia (ALH) if ADH was not also present. Ipsilateral breast cancer was also excluded because the aim of the study was to determine isolated imaging findings associated with the underestimation of these lesions and ensure that the postoperative specimens were independent of other breast lesions. The flow chart of patient inclusion and exclusion is shown in **Figure 1**.

### Data Collection

The clinical features including age, menopausal status, palpability, contralateral tumor, family history of breast cancer, and mammography calcification features were collected from the electronic medical record system. The pathology of CNB was designated ADH according to the ADH diagnostic criteria in the WHO guidelines (19), while the extent of ADH was classified as focal if the pathology report contained “focal ADH,” “focus ADH,” or “single duct of ADH.” In addition, suspected malignancy was coded “yes” in instances where the report contained the statement “suspicious for DCIS” or “suspicious for malignant.” An ADH case was considered “suspected malignancy” if the cytologic and/or architectural features were that of low grade DCIS but either the spectrum of changes present in the core biopsy made it difficult to distinguish if one area definitively qualified for a diagnosis of low grade DCIS or the extent of changes was considered too limited to classify definitively as DCIS on a CNB sample alone (20). The following additional data was also recorded: the co-diagnosis of ADH (adenosis, sclerosing adenosis, papilloma fibroepithelial lesions, and apocrine hyperplasia) and the presence of calcifications within ADH. At final pathological diagnosis, underestimation was defined as cancer found at open excision after a biopsy diagnosis of ADH. In addition, proliferative lesions (neoplasia/atypical lobular hyperplasia, columnar cell change) were classified as borderline lesions (5, 21). The US appearance and category of each lesion were characterized according to the fifth edition of the Breast Imaging Reporting and Data System (BI-RADS) (22). Meanwhile, the different lesion types were classified as mass and non-mass (ductal dilatation, complex cystic lesions, or low echo area) (23). Specific features noted on US examination included the presence or absence of calcifications, and their maximum sizes were measured. Two experienced sonographer (Y.L.C and Y.X.H, with more than 5 years of experience breast ultrasound) who were blinded to the study review the US images, and micro-calcifications with an indistinct oval round or irregular mass, micro-calcifications with a ductal change, and micro-calcifications with a speculated irregular mass were defined as calcification on US.

### Establishment of a Predictive Model and Nomogram

Univariate analysis was used to assess the clinical and demographic parameters, and biopsy, pathological, and US features with the goal of identifying the most relevant predictors of the underestimated risks using Pearson’s Chi-square test or Fisher’s exact test in the training set. The features that had *p*-values of <0.1 after univariate analysis were used to perform multivariate logistic regression with the overarching goal of selecting the most useful variables (*p* < 0.05) *via* stepwise backward selection. In addition, multiple imputation was used in the multivariable logistic analysis to account for missing data (<5%). Twenty-three and 12 imputed data were generated in the training and validation set, respectively. Finally, multivariable logistic regression analysis was applied on the training set to build a nomogram, which is a visual tool for predicting the individualized underestimation risk of patients with biopsy-diagnosed ADH. Two models were built as follows: In the first model, clinical and pathological data were included. In the second model, US features were also added to the predict the upgrade of ADH.

### Predictive Performance and Validation of the Nomogram

The area under curve (AUC) of the receiver operating characteristic (ROC) was used to evaluate the discrimination ability of the nomogram, and followed by calculation of the sensitivity, specificity, and accuracy with 95% confidence intervals (95% CIs). A calibration curve was then plotted to assess the calibration of the nomogram using the Hosmer-Lemeshow goodness-of-fit test. A *P* > 0.05 indicated non-significant deviance from the theoretical perfect calibration. The decision curve analysis was also evaluated. All statistical analysis were conducted using R software (V.3.6.2, http://www.r-project.org) and SPSS v25.0 (SPSS, Inc., IMB Company Chicago, IL, USA).

## Results

### Clinicopathological and US Characteristics

Among the 401 patients included in the training set, 267 (66.6%) were upgraded into malignancy, 126 (31.4%) were benign, and 8 (2.0%) were borderline. Furthermore, 142 (53.2%) of the underestimated cases with malignancy were DCIS, 69 (25.8%) were IDC, 43 (16.1%) were papillary carcinoma, and 3 (1.1%) were invasive lobular carcinoma (ILC). On the other hand, 39 (31.0%) of the benign cases were papilloma, 35 (27.8%) were adenosis, and 10 (7.9%) were fibroadenoma. Of the data in training and validation set, 21 patients had missing data for one or more potential parameters: 8 for family history of breast cancer, 2 for past or present contralateral breast cancer, 9 for mass palpation, 10 for BI-RADS score, and 13 for ADH extent.

The clinicopathological and US characteristics of patients in the underestimated group, non-upgraded group, training set and validation set are listed in **Table 1**. The obtained results indicated that there was no significant difference in the upgrade rate between the training and validation sets (66.6 *vs.* 68.9%, *p* = 0.326). In addition, there were no significant differences in the clinicopathological and US characteristics between the training and validation sets (all *p* > 0.05). Significant differences (*p* < 0.1) were found in age, menopausal status, mass palpation, US diameter, calcifications on US, BI-RADS category, ADH extent, suspected malignancy, and co-diagnosis of ADH.

**Table 1:** Clinicopathological and US factors correlations with upgrades in the training and validation sets.

		Training set			Validation set		
		Upgraded breast cancer		P	Upgraded breast cancer		P
		No (134) (%)	Yes (267) (%)		No (58) (%)	Yes (128) (%)	
Age	<40/>70	35 (26.2)	49 (18.4)	0.015	22 (37.9)	28 (21.8)	0.032
	≤40 ≤ 70	99 (73.8)	218 (81.6)		36 (62.1)	100 (78.1)	
Menopausal status	Pre-	72 (53.7)	117 (61.9)	0.076	25 (44.6)	50 (39.1)	0.516
	Post-	62 (46.3)	147 (70.3)		31 (55.4)	78 (60.9)	
Past or present contralateral breast cancer	No	130 (97.0)	249 (93.3)	0.119	55 (94.8)	118 (93.7)	>0.999
	Yes	4 (3.0)	18 (6.7)		3 (5.3)	8 (6.3)	
Family history of breast cancer	Yes	1 (0.8)	8 (3.0)	0.282	1 (1.7)	3 (2.3)	>0.999
	No	131 (98.2)	255 (97.0)		57 (98.3)	125 (12.0)	
Mass palpation	No	33 (25.4)	35 (13.2)	0.004	17 (29.3)	15 (12.0)	0.006
	Yes	97 (74.6)	230 (86.8)		41 (70.7)	110 (88.0)	
US							
diameter (mm)	<10	16 (11.9)	15 (5.6)	0.031	10 (17.2)	9 (7.0)	0.034
	≥10	118 (88.1)	252 (94.4)		48 (82.8)	119 (72.6)	
Lesion types				0.516			0.488
Mass		109 (81.3)	208 (77.9)		39 (67.2)	93 (72.6)	
Non-mass		25 (18.7)	59 (22.1)		19 (32.8)	35 (27.3)	
Calcifications on US	No	125 (93.3)	145 (54.3)	<0.001	52 (89.7)	71 (55.4)	<0.001
	Yes	9 (6.7)	122 (93.1)		6 (9.5)	57 (90.5)	
Micro-calcification on mammography		(n = 50)	(n = 171)	0.011	(n = 20)	(n = 75)	0.430
	No	27 (67.5)	82 (45.3)		15 (75.0)	36 (48.0)	
	Yes	13 (32.5)	99 (54.7)		5 (20.0)	39 (75.0)	
BI-RADS category				0.058			0.228
	3	3 (2.3)	7 (2.7)		2 (3.5)	3 (2.4)	
	4	128 (97.7)	246 (93.2)		55 (96.5)	117 (93.6)	
	5	0 (0.0)	11 (4.1)		0 (0.0)	5 (4.0)	
CNB feature							
Microscopic calcifications	No	127 (94.8)	256 (95.9)	0.615	56 (96.6)	122 (95.3)	>0.999
	Yes	7 (5.2)	11 (4.1)		2 (3.4)	6 (4.7)	
ADH extent	Focal	123 (95.3)	86 (32.6)	<0.001	51 (91.1)	47 (37.6)	<0.001
	Multifocal	6 (4.7)	178 (67.4)		5 (8.9)	78 (62.4)	
Suspected malignancy	No	125 (93.2)	169 (63.3)	<0.001	55 (94.8)	80 (62.6)	<0.001
	Yes	9 (6.7)	98 (36.7)		3 (5.2)	48 (38.4)	
Co-diagnosis of ADH	No	103 (76.9)	181 (67.8)	0.059	45 (77.6)	82 (64.1)	0.066
	Yes	31 (23.1)	86 (32.2)		13 (22.3)	46 (35.9)	

### Development of the Predictive Nomogram

**Figure 2:**
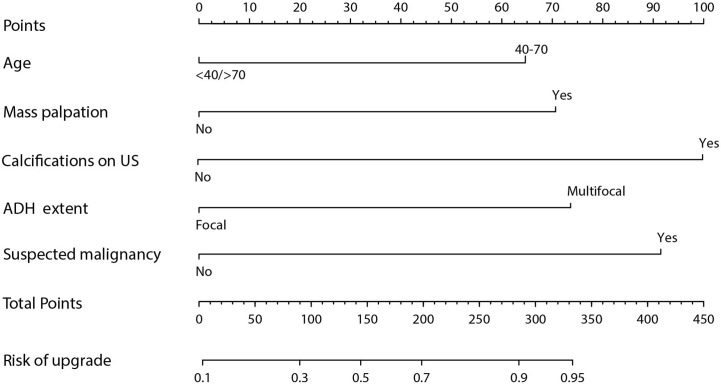
Nomogram based on clinicopathological and US features.

Results obtained after univariate logistic regression analysis indicated that age, menopausal status, mass palpation, calcifications on US, ADH extent, suspected malignant component, and co-diagnosis of ADH were significant risk factors. On the other hand, multivariate logistic regression analysis results showed that the risk factors for upgrade were age (40–70 years *vs.* <40/>70 years, odds ratio 2.739, 95% CI: 1.525–5.672, *p* = 0.01), mass palpation (yes *vs.* no, odds ratio 3.008, 95% CI: 1.624–5.672, *p* < 0.01), calcifications on US (yes *vs.* no, odds ratio 4.752, 95% CI: 2.569–9.276, *p* < 0.01), ADH extent (multifocal *vs.* focal, odds ratio 3.150, 95% CI: 1.951–5.155, *p* < 0.01), and suspicious malignancy (yes *vs.* no, odds ratio 4.162, 95% CI: 2.289–7.980, *p* < 0.01) (**Table 2**). Finally, a nomogram was developed by integrating the independent clinicopathological and US risk factors mentioned above (**Figure 2**).

**Table 2:** Univariate and multivariate logistic regression analyses of factors predictive of upgrading.

Variables	Univariate analysis			Multivariate analysis		
	Odds ratio	95% CI	p	Odds ratio	95% CI	p
Age			0.008			0.010
<40/>70	1			1		
40–70	1.977	1.184–3.291		2.739	1.525–5.672	
Menopausal status			0.015			0.130
No	1			1		
Yes	2.301	1.364–3.885		2.469		
Mass palpation			0.001			<0.001
No	1			1		
Yes	2.301	1.364–3.885		3.008	1.624–5.672	
US diameter (mm)			0.085			–
<10	1					
≥10	1.025	0.997–1.054		–	–	
Calcifications on US			<0.001			<0.001
No	1			1		
Yes	3.567	2.067–6.477		4.752	2.569–9.276	
BI-RADS category			0.588			–
3	1					
4, 5	1.766	0.897–3.332		–	–	
Extent of ADH			<0.001			<0.001
Focal	1			1		
Multifocal	3.071	2.002–4.754		3.150	1.951–5.155	
Suspected malignancy			<0.001			<0.001
No	1			1		
Yes	4.493	2.584–8.262		4.162	2.289–7.980	
Co-diagnosis of ADH			0.001			0.563
No	1			1		
Yes	0.394	0.210–0.763		0.784	0.348–1.824	

### Performance of the Nomogram in the Training and Validation Sets

**Figure 3:**
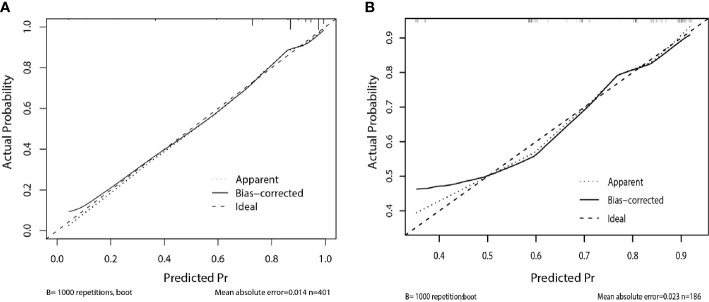
Calibration curve of the nomogram for predicting upgrade to breast cancer: **(A)** in the training set, **(B)** in the validation set. The solid line represents the ideal reference line that predicted ADH upgrade corresponds to the actual outcome, the short-dashed line represents the apparent prediction of nomogram, and the long-dashed line represents the ideal estimation. The prediction performance of the nomogram in training and validation set show closely to observed rates.

**Figure 4:**
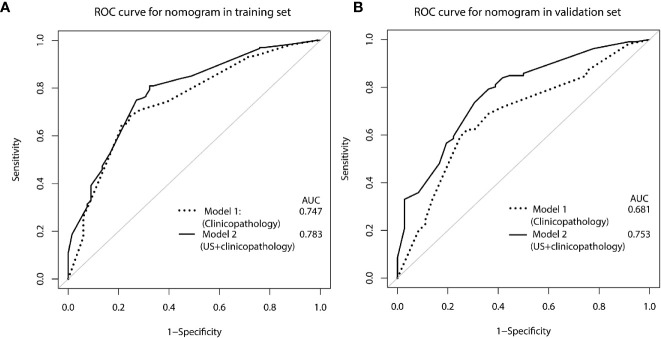
ROC curve for predicting ADH upgrades: **(A)** in the training set, **(B)** in the validation set. Model 1, clinicopathological features; Model 2, US +clinicopathological features.

The calibration curve of the nomogram for the probability of breast cancer upgrade demonstrated good agreement between the predictions and observations in the training and validation sets (*p* > 0.05, **Figure 3**). The Hosmer-Lemeshow test yielded non-significant results in both cohorts (*p* = 0.543 and *p* = 0.565), which suggested that there was no departure from a perfect fit. For predicting the upgrade of ADH, model 1 (clinicopathological features) yield an AUC, a sensitivity, a specificity, and a best cut-off of 0.747 (0.697–0.797), 67.9%, 75.9%, and 0.631, respectively, in the training set, and 0.681 (0.581–0.780), 62.3%, 72.2%, and 0.663 respectively, in the validation set. While model 2 (clinicopathological and US features) yielded an AUC, a sensitivity, a specificity, and a best cut-off of 0.783 (0.736–0.831), 80.9%, 66.2%, and 0.557, respectively, in the training set, 0.753 (0.666–0.841), 80.2%, 63.9%, and 0.574, respectively, in the validation set (**Figure 4**, **Supplementary Table 1**). The total points could be plotted into **Table 3** to conclude the diagnosis values.

**Table 3:** Corresponding mammogram showed grouping and indeterminate micro-calcifications in CC position (B) and MLO position (C).

Points	PPV (%)	NPV (%)	FPR	FNR	SEN (%)	SPE (%)
≤64.7	69.3	78.2	86.5	1.9	98.1	13.4
≤73.6	70.2	80.0	75.9	2.9	97.0	22.4
≤91.5	71.7	78.1	76.1	3.4	96.6	23.8
≤144	82.4	63.3	34.4	19.1	80.9	65.7
≤245	87.0	40.2	97.0	67.4	32.6	90.3
≤309	100	36.0	0	89.1	10.9	100
≤330	100	334.8	0	94.0	59.9	100

PPV, Positive predict value; NPV, Negative predict value; FPR, False positive rate; FNR, False negative rate; SEN, Sensitivity; SPE, Specificity.

### Clinical Application of the Model

**Figure 5:**
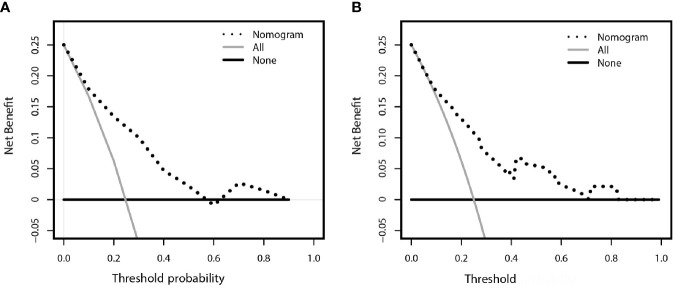
Decision curve analysis comparing the net-benefit of using the nomogram (black clashed line) depicted in **(A)** training set **(B)** validation set. Black solid line: net benefit when all breast cancer patients are considered as not upgrade with ADH; gray solid line: net benefit when all ADH patients are considered as upgraded to breast cancer. The ideal model is the model with highest net benefit at any given threshold.

The decision curve analysis for the constructed nomogram were presented in **Figures 5A, B**. The plot indicated that the model-based decision showed a more net benefit than either the treat-none-patients scheme or the treat-all scheme for the predicted probability thresholds between 0 and 90%.

## Discussion

Despite open-excision being consistently recommended in all the ADH lesions diagnosed by CNB (2), it remains elusive that whether the patients might or might not benefit from the surgery or the surveillance. Therefore, this study developed and validated a US-based nomogram for predicting ADH upgrade in 587 patients (training set, n = 401; validation set, n = 186) under US-guided breast biopsy. The nomogram incorporating two clinical features (age and palpation), one US sign (calcifications), and two pathological features (ADH extent and suspected malignancy). The developed nomogram can be widely applied to facilitate efficient treatment decisions and rational resource allocation, especially in developing countries, because all predicting factors are routinely available before surgery.

The risk factors found in this study are partly similar to those reported in the literature (**Supplementary Table 2**). The difference could be attributed to the sample size and combination of available data. For example, we had 587 cases and 395 events, whereas most published studies had 45 to 422 cases with up to 133 events. With regard to the age of patients, several studies reported various risks in the age category where some found no increase in the risk (24, 25), others reported significant risks in univariable analysis but not in the multivariable analysis (10, 11), and some had significant in multivariable analysis (8). The results obtained in this study indicated that the upgrade rate was higher in middle age years (40–70) and lower at the extremes of age (<40/>70), which was consistent with the findings of another study with a large sample size (9). The higher risk at middle ages can be attributed the fact that the breast gland undergoes tissue remodeling during perimenopausal years, which likely presents a permissive microenvironment for the premalignant epithelium to progress to cancer (9). Similar to the previous studies. We also found that the upgrade was more associated with multifocal ADH than the focal one, which was consistent with previous studies (5, 6, 8, 10, 12, 26). This is probably due to the fact that the foci of ADH may be present at the periphery of DCIS (27). This study also confirmed the report in previous studies that the palpability of lesions is as a risk factor (12, 14, 28). Only a limited number of studies have reported that suspected malignancy a high risk for upgrading biopsies with a suspected component (20).

**Figure 6:**
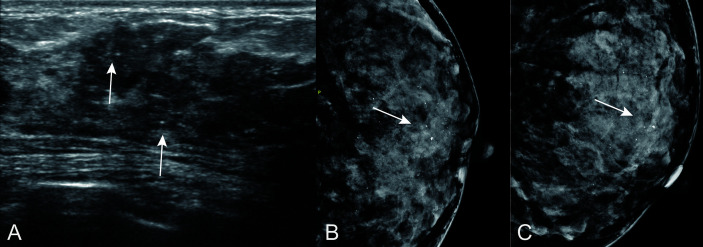
Calcifications on US in patients with ADH on CNB. A 44 years old woman diagnosed with sclerosing adenosis at final pathologic examination. **(A)** US showed sparse calcifications (arrow) at hypoechoic hypoechoic non-mass of breast lesion. Corresponding mammogram showed regional microcalcifications in CC position **(B)** and MLO position **(C)**.

**Figure 7:**
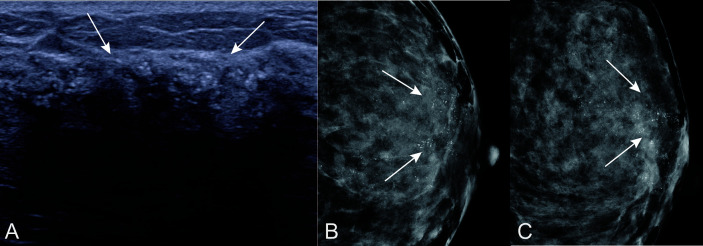
Calcifications on US in patients with ADH on CNB. A 55 years old woman diagnosed with DCIS at final pathologic examination. **(A)** US showed calcifications (arrow) around with hypoechoic non-mass of breast lesion. Corresponding mammogram showed grouping and indeterminate microcalcifications in CC position **(B)** and MLO position **(C)**.

Despite the clinical and pathological factors, we found that US features may help predict the possibility of breast cancer upgrade. Specifically, calcifications on the US were more discriminative than any other risk factor in the model, with a high positive predictive value because they were present in 93% of cases having positive surgical excisions (**Figures 6** and **7**). This association between the likelihood of malignancy and calcifications on mammography is not unexpected, because several previous studies reported that the extent of calcifications clusters (>15mm) is a determinant feature for malignancy on mammography, and incomplete removal of calcifications was considered to be correlated with the upgrade of ADH (12, 25, 29). Although mammography is the standard method for evaluating breast calcification, however, it is expensive to perform in developing countries. It is worth noting that US is also able to detect most microcalcifications (with a maximum diameter of 1 mm) that correlates well with mammography (30–32). To our knowledge, calcifications on US has never been demonstrated in the prediction of ADH before. One study observed that calcifications was associated with upgrade rate under ultrasound-guided CNB, but whether the calcifications on US was the same as it on MG was not clear (12). It has been though that breast tumor cells acquired osteoblast-like phenotype through epithelial-mesenchymal transition and drive pathophysiological microcalcifications, but little has been understood about the precise underlying molecular mechanism (33). A higher specificity (86.9%) has been reported in a nomogram (11) incorporating nine clinical, radiological, and pathological variables, but with a relatively small sample size (n = 203) without a validation set. On the other hand, Ko et al. (12) proposed a scoring system based on clinical, imaging, and pathological features with a diagnostic power of 0.903. This system was externally validated by Bendifallah et al. (13), which resulted in a low reproducibility of 0.510 and specificity of 0.22.

The nomogram we developed could support the clinical decision of ADH diagnosed on CNB. For example, point 91.5 indicated a FNR less than 5%, this might indicate a lower risk for upgraded according to the consensus that overall underestimation rates of ADH should not exceed 5% for invasive cancer and 10% for DCIS (2). Corresponding to the predictive model with specificity of 0.639, use of the nomogram could have avoided surgery in more than 60% of the patients with finally confirmed benign diagnosis. On the other hand, the upgrade rate of 66.6% (267/401) in our results indicates that excision of the ADH is essential for finding already existing breast cancers. Furthermore, we found that about one-fourth of the cancer were IDC, which was in line with or somewhat higher than the results reported in a previous study where one-fifth of the upgraded lesions were invasive breast cancer and non-low-grade DCIS (34). This might be related to the heterogeneity of breast cancer that DCIS is always co-existing with IDC (35). The higher rate of IDC in our study may indicate that ADH lesions were obtained neighboring malignancy with CNB (21). The necessity of the excision procedure was further strengthened by another study where multiple surgeries were reported in 29% of the patients diagnosed with ADH, including re-excision and mastectomy, to achieve clear margin in extensive DCIS cases (34).

Clinically, more breast lesion types are detectable and under direct vision by US-guided biopsy. MRI is superior to US and mammography because it has a high sensitivity in breast imaging diagnosis. However, the cost of MRI remains a great economic burden and not all the women can tolerate MRI scanning procedure. Application of the nomogram model the developed in this study can help high-risk patients receive prompt treatment with a valuable decrease in patient waiting anxiety. However, the potential for reducing costs for low-risk patients should be explored in a dedicated analysis considering long-interval follow-up recommendations and additional imaging supporting. Combining artificial intelligence and molecular methodology in a large number of cases, through collaborative and, multicenter studies has the potential to advance knowledge in this field and assist in appropriate design of trials of treatment decision for women diagnosed with ADH using CNB (36).

This study had the following limitations: Firstly, the nomogram was established and validated based on retrospective data. Therefore, subsequent prospective randomized trials should be conducted to determine whether the nomogram model improves current patient stratification approaches for clinical decision-making and the corresponding prognosis. Secondly, the calcifications detected by US were not confirmed by mammography, which is considered to have a more diagnostic value. Further studies using large datasets that compare and correlate predictive radiological features in multiple imaging guidance methods, including stereotactic (mammographic) guidance, US guidance, and MRI guidance, may help to facilitate the development of an individualized model as a promising tool for clinical use.

## Conclusion

This study developed an easy-to-use nomogram model that incorporates clinicopathological and US features to predict the upgrade of ADH on CNB. Application of the nomogram model can provide information for clinical procedure planning and potentially increase clinical efficiency.

## Data Availability Statement

The datasets used and analysed during the current study are readily available from the corresponding author upon reasonable request.

## Ethics Statement

The studies involving human participants were reviewed and approved by the Ethics Committees of Fudan University Cancer Center. The patients/participants provided their written informed consent to participate in this study. Written informed consent was obtained from the individual(s) for the publication of any potentially identifiable images or data included in this article.

## Author Contributions

Y-XH and S-CZ contributed to the conception and design of the study, data analysis and interpretation, and drafting the manuscript. Y-LC, KZ, and S-PL collected and analyzed US and pathological data,while CC and J-PS contributed to the acquisition and assembly of data. All authors contributed to the article and approved the submitted version.

## Funding

This work was supported by the Science and Technology Commission of Shanghai Municipality (18411967400, 17411953400), Shanghai Municipal Commission of Health and Family Planning (20174Y0011), Shanghai Anticancer Association EYAS PROJECT (SACA-CY1C06), and Fudan University Shanghai Cancer Center Special Fund for Iconography (YX201804).

## Conflict of Interest

The authors declare that the research was conducted in the absence of any commercial or financial relationships that could be construed as a potential conflict of interest.
