# Supplementary material for: Development and Validation of a Simple-to-Use Nomogram for Predicting the Upgrade of Atypical Ductal Hyperplasia on Core Needle Biopsy in Ultrasound-Detected Breast Lesions
Source: Front Oncol. 2021 Mar 31;10:609841. doi: 10.3389/fonc.2020.609841 (PMC8044403; doi:10.3389/fonc.2020.609841)
Supplement: Supplementary file 1 [file Table_1.pdf]

Performance of predictive models with different features

	AUC	SEN	SPE	NPV	PPV
Model 1_t	0.747	0.679	0.759	0.540	0.850
Model 2_t	0.783	0.809	0.662	0.638	0.834
Model 1_v	0.681	0.623	0.722	0.393	0.868
Model 2_v	0.753	0.802	0.639	0.500	0.864

Model 1: incorporating clinical and pathological features.

Model 2: incorporating clinical and pathological features.

t: training set; v: validation set
