# Supplementary material for: Development and Validation of a Simple-to-Use Nomogram for Predicting the Upgrade of Atypical Ductal Hyperplasia on Core Needle Biopsy in Ultrasound-Detected Breast Lesions
Source: Front Oncol. 2021 Mar 31;10:609841. doi: 10.3389/fonc.2020.609841 (PMC8044403; doi:10.3389/fonc.2020.609841)
Supplement: Supplementary file 2 [file Table_2.pdf]

Review of the factors associated with the upgrade of ADH diagnosed on CNB

Variables[a,b]	Significance of potential risk factors, as described in literature[c,d]		
	No	Yes, univariable	Yes, multivariable
Age[d]	**1**, 2, **3**, 4, 5, 6, 7, 8, 9, 10	11, **12**, **13**, 14, **15**, 16, 17	11, **12**, 14, **15**, 17
History of breast cancer[d]	**1**, 2, **3**, 4, 5, 8, 9, **12**, 16	7	
Family history[d]	**1**, 2, **3**, 4, 5, 7, 8, 9, **12**, **13**		
Palpability	**3**, 4, 16	14, **15**	9, 14, **15**
Lesion type on imaging	**1**, 4, 7	**13**	5, **13**
Lesion size	4, 9, 16	**1**, 7, 8, 10, **12**, 14, **15**, 17, 18	5, 8, **12**, 14, **15**, 17
BI-RADS score	**3**, 7, 10, **13**, 17		4, 5, 9
Calcifications on mammography/imaging	**3**, 6, 10, 16, 18	14, **15**, 17	9, 14, **15**
Other mammography features	8, 18	2, 17	2, 9, 17
Type of biopsy gauge	5, **12**, **13**, 19	**1**, 2, 16, 20	2
Residual mammographic calcifications	8, 16, 21	**1**, 6, 7, 18	**1**
Number of cores[d]	6, 7, 11, **12**, 16, 18	**1**, 10	
Number of involved cores	7, **12**	6, **13**	**13**
Extent of ADH[d]	**3**, 10, **15**, 16	**1**, 2, 6, 8, 11, **12**, **13**, 14, 21, 22	**1**, 2, 8, 11, **12**, 14
Calcifications on ADH[d]	6, 11,**12**	**13**	
Size of focus	22	6, **13**	**13**

Suspicious malignancy		**1**, 22	**1**, 22
ADH type[d,e]	**12**, **13**, 22	**1**, 6, 11	11
Co-diagnosis of ADH	5, **12**, 18	2, 10, 11	2, 10, 11

[a]Variables in italics are variables that were analysed in this study.

[b]Variables that were analysed but were not statistically significant in any study were race[13], menopausal status[3, 5, 13], hormone therapy[3,13], concurrent lobular neoplasia[1, 13], and CNB imaging modality[1, 12, 18].

[c]Listed are 22 studies with at least 20 cases of upgrade.

[d]The categories of these variables were not uniformly defined between studies.

[e]ADH type: cribriform pattern[10], micropapillary pattern[10, 12], solid pattern[10].

[g]Reference in bold are of the fie studies that developed a prediction model.

Reference

1. Pena, A, Shah, SS, Fazzio, RT, Hoskin, TL, Brahmbhatt, RD, Hieken, TJ, et al. Multivariate model to identify women at low risk of cancer upgrade after a core needle biopsy diagnosis of atypical ductal hyperplasia. *Breast Cancer Res Treat.* (2017) 164, 295-304. doi: 10.1007/s10549-017-4253-1
2. Deshaies, I, Provencher, L, Jacob, S, Cote, G, Robert, J, Desbiens, C, et al. Factors associated with upgrading to malignancy at surgery of atypical ductal hyperplasia diagnosed on core biopsy. *Breast.* (2011) 20, 50-5. doi: 10.1016/j.breast.2010.06.004
3. Bendifallah, S, Defert, S, Chabbert-Buffet, N, Maurin, N, Chopier, J, Antoine, M, et al. Scoring to predict the possibility of upgrades to malignancy in atypical ductal hyperplasia diagnosed by an 11-gauge vacuum-assisted biopsy device: an external validation study. *Eur J Cancer.* (2012) 48, 30-6. doi: 10.1016/j.ejca.2011.08.011
4. Hsu, HH, Yu, JC, Hsu, GC, Yu, CP, Chang, WC, Tung, HJ, et al. Atypical ductal hyperplasia of the breast diagnosed by ultrasonographically guided core needle biopsy. *Ultraschall Med.* (2012) 33, 447-54. doi: 10.1055/s-0029-1245877
5. Hodorowicz-Zaniewska, D, Brzuszkiewicz, K, Szpor, J, Kibil, W, Matyja, A, Dylag-Trojanowska, K, et al. Clinical predictors of malignancy in patients diagnosed with atypical ductal hyperplasia on vacuum-assisted core needle biopsy. *Wideochir Inne Tech Maloinwazyjne.* (2018) 13, 184-91. doi: 10.5114/wiitm.2018.73528

6. Wagoner, MJ, Laronga, CAcs, G. Extent and histologic pattern of atypical ductal hyperplasia present on core needle biopsy specimens of the breast can predict ductal carcinoma in situ in subsequent excision. *Am J Clin Pathol.* (2009) 131, 112-21. doi: 10.1309/AJCPGHEJ2R8UYFGP
7. Jackman, RJ, Birdwell, RLIkeda, DM. Atypical ductal hyperplasia: can some lesions be defined as probably benign after stereotactic 11-gauge vacuum-assisted biopsy, eliminating the recommendation for surgical excision? *Radiology.* (2002) 224, 548-54. doi: 10.1148/radiol.2242011528
8. Forgeard, C, Benchaib, M, Guerin, N, Thiesse, P, Mignotte, H, Faure, C, et al. Is surgical biopsy mandatory in case of atypical ductal hyperplasia on 11-gauge core needle biopsy? A retrospective study of 300 patients. *Am J Surg.* (2008) 196, 339-45. doi: 10.1016/j.amjsurg.2007.07.038
9. Co, M, Kwong, AShek, T. Factors affecting the under-diagnosis of atypical ductal hyperplasia diagnosed by core needle biopsies - A 10-year retrospective study and review of the literature. *Int J Surg.* (2018) 49, 27-31. doi: 10.1016/j.ijsu.2017.11.005
10. Farshid, G, Edwards, S, Kollias, JGill, PG. Active surveillance of women diagnosed with atypical ductal hyperplasia on core needle biopsy may spare many women potentially unnecessary surgery, but at the risk of undertreatment for a minority: 10-year surgical outcomes of 114 consecutive cases from a single center. *Mod Pathol.* (2018) 31, 395-405. doi: 10.1038/modpathol.2017.114
11. Chen, LY, Hu, J, Tsang, JYS, Lee, MA, Ni, YB, Chan, SK, et al. Diagnostic upgrade of atypical ductal hyperplasia of the breast based on evaluation of histopathological features and calcification on core needle biopsy. *Histopathology.* (2019) 75, 320-28. doi: 10.1111/his.13881
12. Salagean, ED, Slodkowska, E, Nofech-Mozes, S, Hanna, W, Parra-Herran, CLu, FI. Atypical ductal hyperplasia on core needle biopsy: Development of a predictive model stratifying carcinoma upgrade risk on excision. *Breast J.* (2019) 25, 56-61. doi: 10.1111/tbj.13155
13. Khoury, T, Chen, X, Wang, D, Kumar, P, Qin, M, Liu, S, et al. Nomogram to predict the likelihood of upgrade of atypical ductal hyperplasia diagnosed on a core needle biopsy in mammographically detected lesions. *Histopathology.* (2015) 67, 106-20. doi: 10.1111/his.12635
14. Ko, E, Han, W, Lee, JW, Cho, J, Kim, EK, Jung, SY, et al. Scoring system for predicting malignancy in patients diagnosed with atypical ductal hyperplasia at ultrasound-guided core needle biopsy. *Breast Cancer Res Treat.* (2008) 112, 189-95. doi: 10.1007/s10549-007-9824-0
15. Kim, J, Han, W, Go, EY, Moon, HG, Ahn, SK, Shin, HC, et al. Validation of a scoring system for predicting malignancy in patients diagnosed with atypical ductal hyperplasia using an ultrasound-guided core needle biopsy. *J Breast Cancer.* (2012) 15, 407-11. doi: 10.4048/jbc.2012.15.4.407
16. Mcghan, LJ, Pockaj, BA, Wasif, N, Giurescu, ME, Mccullough, AEGray, RJ. Atypical ductal hyperplasia on core biopsy: an automatic trigger for excisional biopsy? *Ann Surg Oncol.* (2012) 19, 3264-9. doi: 10.1245/s10434-012-2575-0
17. Hong, ZJ, Chu, CH, Fan, HL, Hsu, HM, Chen, CJ, Chan, DC, et al. Factors predictive of breast cancer in open biopsy in cases with atypical ductal hyperplasia diagnosed by ultrasound-

guided core needle biopsy. *Eur J Surg Oncol.* (2011) 37, 758-64. doi: 10.1016/j.ejso.2011.06.014

18. Williams, KE, Amin, A, Hill, J, Walter, C, Inciardi, M, Gatewood, J, et al. Radiologic and Pathologic Features Associated With Upgrade of Atypical Ductal Hyperplasia at Surgical Excision. *Acad Radiol.* (2019) 26, 893-99. doi: 10.1016/j.acra.2018.09.010
19. Eby, PR, Ochsner, JE, Demartini, WB, Allison, KH, Peacock, SLehman, CD. Is surgical excision necessary for focal atypical ductal hyperplasia found at stereotactic vacuum-assisted breast biopsy? *Ann Surg Oncol.* (2008) 15, 3232-8. doi: 10.1245/s10434-008-0100-2
20. Darling, ML, Smith, DN, Lester, SC, Kaelin, C, Selland, DL, Denison, CM, et al. Atypical ductal hyperplasia and ductal carcinoma in situ as revealed by large-core needle breast biopsy: results of surgical excision. *AJR Am J Roentgenol.* (2000) 175, 1341-6. doi: 10.2214/ajr.175.5.1751341
21. Kohr, JR, Eby, PR, Allison, KH, Demartini, WB, Gutierrez, RL, Peacock, S, et al. Risk of upgrade of atypical ductal hyperplasia after stereotactic breast biopsy: effects of number of foci and complete removal of calcifications. *Radiology.* (2010) 255, 723-30. doi: 10.1148/radiol.09091406
22. Allison, KH, Eby, PR, Kohr, J, Demartini, WBLehman, CD. Atypical ductal hyperplasia on vacuum-assisted breast biopsy: suspicion for ductal carcinoma in situ can stratify patients at high risk for upgrade. *Hum Pathol.* (2011) 42, 41-50. doi: 10.1016/j.humpath.2010.06.011
